# PD-L1 expression as predictive biomarker in patients with NSCLC: a pooled analysis

**DOI:** 10.18632/oncotarget.7582

**Published:** 2016-02-22

**Authors:** Francesco Passiglia, Giuseppe Bronte, Viviana Bazan, Clara Natoli, Sergio Rizzo, Antonio Galvano, Angela Listì, Giuseppe Cicero, Christian Rolfo, Daniele Santini, Antonio Russo

**Affiliations:** ^1^ Section of Medical Oncology, Department of Surgical, Oncological and Oral Sciences, University of Palermo, Palermo, Italy; ^2^ Department of Medical, Oral and Biotechnological Sciences, University “G. D'Annunzio”, Chieti, Italy; ^3^ Phase I- Early Clinical Trials Unit, Oncology Department and Multidisciplinary Oncology Center Antwerp (MOCA), Antwerp University Hospital, Edegem, Belgium; ^4^ Medical Oncology Department, Campus Biomedico, University of Rome, Rome, Italy

**Keywords:** PD-L1, predictive biomarker, immunotherapy, anti-PD1/PD-L1 MoAbs, NSCLC

## Abstract

**Background:**

Clinical trials of immune checkpoints modulators, including both programmed cell death-1 (PD-1) and programmed cell death-ligand 1 (PD-L1) inhibitors, have recently shown promising activity and tolerable toxicity in pre-treated NSCLC patients. However the predictive role of PD-L1 expression is still controversial. This pooled analysis aims to clarify the association of clinical objective responses to anti PD-1/PD-L1 monoclonal antibodies (MoAbs) and tumor PD-L1 expression in pre-treated NSCLC patients.

**Methods:**

Data from published studies, that evaluated efficacy and safety of PD-1/PD-L1 inhibitors in pre-treated NSCLC patients, stratified by tumor PD-L1 expression status (immunohistochemistry, cut-off point 1%), were collected by searching in PubMed, Cochrane Library, American Society of Clinical Oncology, European Society of Medical Oncology and World Conference of Lung Cancer, meeting proceedings. Pooled Odds ratio (OR) and 95% confidence intervals (95% CIs) were calculated for the Overall Response Rate (ORR) (as evaluated by Response Evaluation Criteria in Solid Tumors, version 1.1), according to PD-L1 expression status.

**Results:**

A total of seven studies, with 914 patients, were eligible. Pooled analysis showed that patients with PD-L1 positive tumors (PD-L1 tumor cell staining ≥1%), had a significantly higher ORR, compared to patients with PD-L1 negative tumors (OR: 2.44; 95% CIs: 1.61-3.68).

**Conclusions:**

PD-L1 tumor over-expression seems to be associated with higher clinical activity of anti PD-1/PD-L1 MoAbs, in pre-treated NSCLC patients, suggesting a potential role of PD-L1 expression, IHC cut-off point 1%, as predictive biomarker for the selection of patients to treat with immune-checkpoint inhibitors.

## INTRODUCTION

Cancer immunotherapy is emerging as a very promising therapeutic strategy for several solid tumors, including non-small cell lung cancer (NSCLC). Differently from other treatment approaches directed against the tumor, such as chemotherapy or targeted therapy [1-6], targeting the immune system offers the potential for durable activity and long-term survival outcomes, regardless of tumor's histological subtype or mutation status, with a unique, tolerable, toxicity profile. Among the different immunotherapeutic strategies under clinical investigation in NSCLC, the blockade of inhibitory immune-checkpoints with monoclonal antibodies (MoAbs), is currently considered the most promising approach, promoting the immune-response against cancer cells [7-9]. Programmed cell death protein-1 (PD-1) is a checkpoint receptor expressed on the surface of activated T-cells, as well as on B-cells and natural killers (NK) [10], binding its natural ligands, PD-L1 and PD-L2, which may be expressed by both stromal and tumor cells [11]. The PD-1/PD-L1 axis is an inhibitory signaling pathway, causing T-cells exhaustion and inactivation, to prevent autoimmune response [11-13]. However it represents also an important mechanism of immune-escape, co-opted by the tumor cells to limit T-cells activity in the tumor microenvironment during the late-stage of the “immune-editing process” [14]. An improved understanding of cancer immunology has led to the development of several MoAbs which are able to revert a non-efficient or suppressed immune-response by the blockade of the PD-1/PD-L1 axis [15, 16]. There are two different classes of MoAbs: the anti-PD-1 MoAbs, Nivolumab and Pembrolizumab are fully human and humanized, respectively, IgG4 MoAbs, blocking the binding between PD-1 receptor and its natural ligands, PD-L1 and PD-L2; the anti-PD-L1 MoAbs Atezolizumab, Durvalumab, and Avelumab are IgG1 isotypes with genetically modified Fc fragments, which block the PD-L1 and prevent its interaction with PD-1 receptor [17, 18]. All these MoAbs have shown a very promising activity in early phase I trials, reaching an overall response rates (ORR) of about 20%, in a heavily pre-treated and unselected NSCLC population [19-22]. Most of such responses occur relatively early, about 50% within eight weeks of treatment, and may be maintained for a long time [19]. These encouraging data have been recently confirmed by two prospective, randomized, phase III trials, comparing Nivolumab vs Docetaxel, in both squamous and non-squamous, advanced NSCLC, after prior chemotherapy-regimens failure [23, 24]. Even more exciting was the overall survival (OS) benefit obtained with Nivolumab in this setting of patients, leading to the approval of the first anti-PD-1 MoAb, by the Food and Drug Administration (FDA), for the second-line treatment of squamous NSCLC. A relationship between PD-L1 expression on tumor cells and ORR has been first suggested by the phase I study of Topalian et al. [25]. In such study among 42 patients with different solid tumors evaluated with immunohistochemical analysis, none of those with PD-L1 negative tumor obtained an ORR, while about one third of patients with PD-L1 positive tumors had a clinical response. Since then, almost all clinical studies of immune-checkpoint inhibitors in NSCLC investigated the potential correlation between tumor PD-L1 expression and anti-PD-1/PD-L1 MoAbs activity/efficacy [19-24, 26-30], in order to validate PD-L1 expression as predictive biomarker. The majority of such studies have shown that PD-L1 over-expression is associated with significantly higher ORR in pre-treated NSCLC population [19, 20, 22, 24, 27-29], while some other studies have not found a significant association [21, 23, 26]. Although different immunohistochemistry (IHC) cut-off points, ranging from 1% to 50%, have been used to define the PD-L1 positivity in tumor specimens, the results of the phase III CheckMate 057 study [24] have recently shown that PD-L1 (IHC, cut-off point 1%) significantly correlated with ORR, progression free survival (PFS) and overall survival (OS), in pre-treated NSCLC patients. These data suggested PD-L1 at the lowest expression level (IHC, cut-off point 1%) as the best cut-off, allowing to include all patients who may really benefit from these therapies. The aim of this pooled-analysis is to combine and analyze simultaneously all the studies reporting the ORR of pre-treated NSCLC patients receiving anti PD1/PD-L MoAbs, stratified according to the PD-L1 expression status (IHC, cut-off point 1%), in order to provide a more precise estimation of the predictive role of PD-L1 expression in NSCLC.

## RESULTS

**Figure 1:**
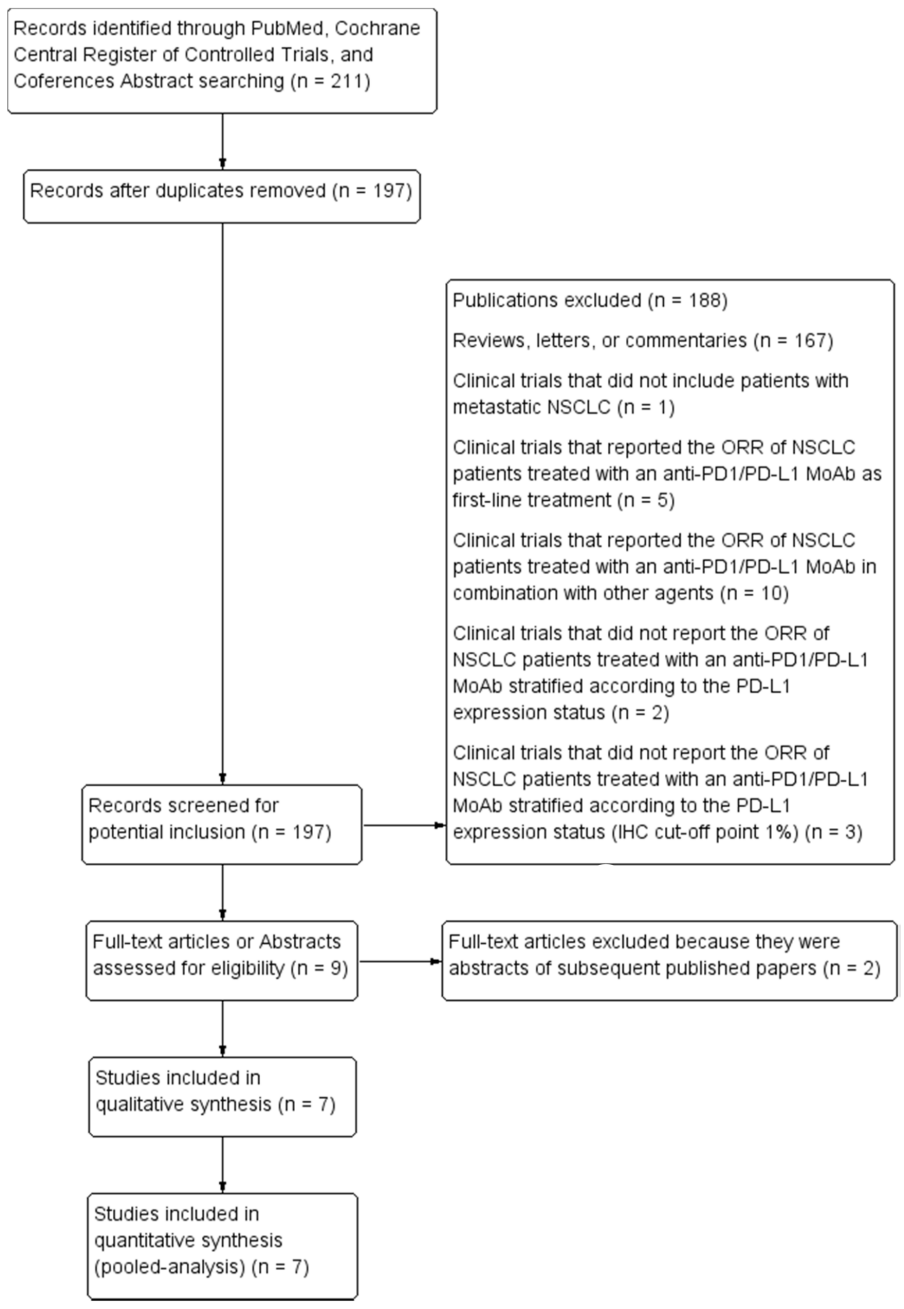
Flow-chart of studies selection

Our search, according to the aforementioned criteria, performed in October 2015, identified 211 publications. Among these, after a careful selection procedure, only seven studies (914 patients) met our inclusion criteria and were included in the pooled-analysis (Figure 1) [20, 21, 23, 24, 26, 27, 30]. Three of these were early phase I studies, reporting the updated results of the immune-checkpoint inhibitors, Pembrolizumab [20], Atezolizumab [21], and Avelumab activity [30], in pre-treated NSCLC patient cohorts. In particular, the KEYNOTE-001 was a modern, large phase I study, including about 500 NSCLC patients treated with Pembrolizumab, which has shown an ORR of about 20% and an OS of about 12 months in the overall population, even higher among the patients with an increased tumor PD-L1 expression [20]. Two were phase II studies: a single arm trial of Nivolumab in patients with advanced refractory squamous NSCLC [26], and the randomized phase II trial comparing Atezolizumab vs docetaxel in 287 pre-treated NSCLC patients, whose interim results have recently shown a significant benefit in favour of Atezolizumab, correlating with an increasing tumor PD-L1 expression [27]. Two randomized, phase III studies, comparing Nivolumab vs docetaxel, in pre-treated, both squamous [23] and non-squamous [24] NSCLC patients, have both shown a significant improvement of the ORR and OS in the overall population treated with Nivolumab, reporting opposite results in the PD-L1 expression analysis. Indeed, the CheckMate 057 study demonstrated a strong predictive value of tumor PD-L1 expression in non-squamous NSCLC [24], while no significant association between Nivolumab benefit and PD-L1 expression has been found in patients with squamous histology in the CheckMate 017 trial [23]. In the included studies, sample sizes of the analyzed population ranged from 53 [21] to 231 [24], while the percentage of PD-L1 positive tumors, IHC cut-off point 1%, ranged from 50% [21] to 85% [20]. All these studies analyzed the ORR of pre-treated NSCLC patients stratified by tumor PD-L1 expression status (immunohistochemistry, cut-off point 1%). A detailed description of the selected studies is reported in the Table 1.

**Table 1:** Characteristics of the trials included in the pooled-analysis

Study (reference)	Drug	Detection (cut-point)	ORR (PD-L1+) n.*(%)	ORR (PD-L1-) n.*(%)	PFS (PD-L1+) m. (95% CI)	PFS (PD-L1-) m. (95% CI)	OS (PD-L1+) m. (95% CI)	OS (PD-L1-) m. (95% CI)
Rizvi et al. 2015 (phase II) (26)	Nivolumab	IHC (1%)	9/45 (20)	4/31 (13)	N.A	N.A	N.A	N.A
Brahmer et al. 2015 (phase III) (23)	Nivolumab	IHC (1%)	11/63 (17)	9/54 (17)	3.3	3.1	9.3	8.7
Borghaei et al. 2015 (phase III) (24)	Nivolumab	IHC (1%)	38/123 (31)	10/108 (9)	4.2	2.1	17.2	9.4
Garon et al. 2015 (phase I) (20)	Pembrolizumab	IHC (1%)	37/134 (27)	2/22 (9)	N.A	N.A	N.A	N.A
Rizvi et al. 2014 (phase I) (21)	Atezolizumab	IHC (1%)	8/26 (31)	5/26 (20)	N.A	N.A	N.A	N.A
Spira et al. 2015 (phase II) (27)	Atezolizumab	IHC (1%)	17/93 (18)	4/51 (8)	3.3	1.9	N.A	N.A
Gulley et al. 2015 (phase I) (30)	Avelumab	IHC (1%)	17/118 (14)	2/20 (10)	3	1.4	N.A	N.A

ORR, Overall Response Rate; PD-L1; Programmed death-ligand 1; IHC, immunohistochemistry; n., number; m., months; N.A, not available.

* The number of patients reported corresponds to the number of patients evaluable

**Figure 2:**
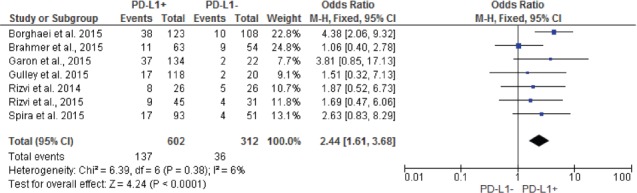
Forest plot showing odds ratio for overall response rate to anti-PD-1/PD-L1 monoclonal antibodies according to the tumor PD-L1 expression status, in pre-treated NSCLC patients

**Figure 3:**
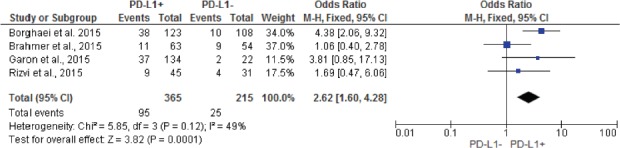
Forest plot showing odds ratio for overall response rate to anti-PD-1 monoclonal antibodies according to the tumor PD-L1 expression status, in pre-treated NSCLC patients

**Figure 4:**
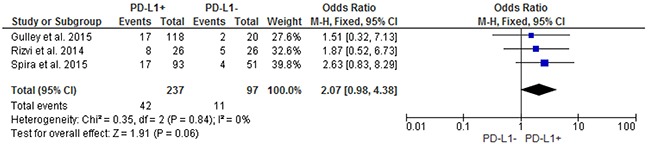
Forest plot showing odds ratio for overall response rate to anti-PD-L1 monoclonal antibodies according to the tumor PD-L1 expression status, in pre-treated NSCLC patients

**Figure 5:**
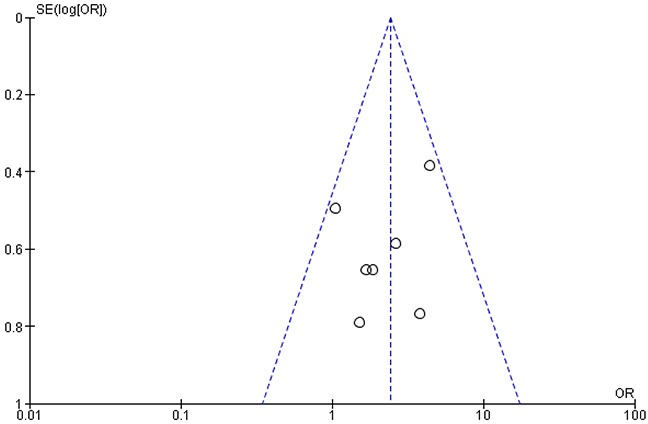
Funnel plot of odds ratio (OR) for response rate (RR) according to the PD-L1 expression status in pre-treated NSCLC patients receiving anti-PD-1/PD-L1 treatments Each study is represented by one circle- the vertical line represents the pooled effect estimate.

Pooled analysis showed that patients with PD-L1 positive tumors (PD-L1 tumor cell staining ≥1%) had a significantly higher ORR compared to patients with PD-L1 negative tumors (OR: 2.44; 95% CIs: 1.61-3.68) (Figure 2). A significant difference in activity by PD-L1 status was confirmed for anti-PD1 MoAbs (OR: 2.62; 95% CIs: 1.60-4.28) (Figure 3), with a no significant trend for anti-PD-L1 MoAbs (OR: 2.07; 95% CIs: 0.98-4.38) (Figure 4). The pooled OR for ORR was calculated using fixed-effect model, because of non-significant heterogeneity between treatment effects (Q-test: P: 0.38). Publication bias have not been found either by Begg's and Egger's tests for ORR (P: 0.5 and P: 0.27, respectively), according to the PD-L1 expression status (Figure 5).

## DISCUSSION

This pooled analysis included seven studies which evaluated the ORR of patients with NSCLC treated with anti PD1/PD-L1 MoAbs who received prior chemotherapy regimens stratified according to the tumor PD-L1 expression status (IHC, cut-off point 1%). The results of this work have shown that tumor PD-L1 positivity (PD-L1 tumor cell staining ≥1%) is associated with significantly higher ORR, suggesting a potential role of tumor PD-L1 over-expression as predictive biomarker for clinical setting. After the recent approval of Nivolumab by FDA for the second-line treatment of squamous NSCLC, searching for predictive biomarkers has become an urgent issue for clinical research. A validated biomarker would allow to select those patients who could benefit more from immune-therapies, sparing the others from ineffective high-cost treatments, as well as futile immune-related toxicities. In this scenario, tumor PD-L1 expression represents the predictive biomarker most evaluated in clinical studies. Although the majority of such studies suggested a significant association between tumor PD-L1 expression and anti-PD-1/PD-L1 MoAbs activity [20, 24, 27], the two largest phase III randomized CheckMate 017 and 057 trials have shown opposite results. Indeed the CheckMate 017 study of Nivolumab in squamous NSCLC did not report any difference in terms of efficacy according to the tumor PD-L1 expression status [23], whereas the CheckMate 057 has recently shown that ORR nearly tripled and median OS nearly doubled with Nivolumab vs Docetaxel in pre-treated patients with PD-L1 positive non-squamous NSCLC [24]. Such data suggested that PD-L1 has a predictive value limited to the adenocarcinoma, likely influencing also the results of our analysis. An individual patients data analysis by histology would be useful to determine if the predictive role of PD-L1 expression in NSCLC is histology-driven, but unfortunately we didn't have such informations. Another major issue emerging from the studies regards the heterogeneity of the IHC cut-offs, ranging from >1% to >50%, used to define the PD-L1 positivity in tumor specimens. Indeed PD-L1 has shown to be predictive of Nivolumab activity at different cut offs of 1%, 5% and 10% [24], while in the more recent KEYNOTE-001 trial of pembrolizumab in advanced NSCLC, patients' survival significantly differed between patients with PD-L1 expression >50% in comparison with patients with a PD-L1 <50% [20]. The multitude of the detection methods by different PD-L1 IHC MoAbs used in the included studies, has further complicated the interpretation of the biomarker data analysis as well as their clinical applicability. The International Association for the Study of Lung Cancer (IASLC) Pathology Committee is currently working on this field in order to standardize and validate a reproducible IHC test for PD-L1 assessment [31]. According to the CheckMate 057 results [24], our work also demonstrated that tumor PD-L1 expression at the lowest IHC cut-off of 1%, significantly correlated with anti-PD-1/PD-L1 MoAbs activity in pre-treated NSCLC patients, confirming its potential role as predictive biomarker for clinical use. The non-significant trend observed for anti-PD-L1 MoAbs is likely due to the lower number of patients included in the subgroup analysis. Similarly to other studies our work also demonstrated that more than 10% of PD-L1 negative patients received also benefit by anti PD-1/ PD-L1 therapies, suggesting that tumor PD-L1 expression could not be able to predict the overall immunotherapy benefit in NSCLC [32, 33]. Of course, the ORR (WHO, RECIST) may not be considered as the best endpoint to assess the predictive value of tumor PD-L1 expression. Differently from oncogene drivers, such as EGFR and EML4-ALK, which are associated with high response rates to targeted therapies (1-4), the predictive power of tumor PD-L1 expression should be evaluated on the basis of long-term survival outcomes associated with these treatments. To date most of such outcomes are still immature, although preliminary OS for some of the anti-PD1/PD-L1 inhibitors seems to be associated with tumor PD-L1 status [20, 24]. A retrospective analysis presented at the last WCLC has shown that patients with NSCLC who achieved a best response (CR or PR) in the single arm study (CA209063) of Nivolumab had the longest survival, suggesting the ORR as a reliable surrogate endpoint of OS in patients receiving immunotherapy [34]. In addition patients whose best response was SD or PD according to RECIST and continued Nivolumab beyond PD had longer survival compared to patients with no treatment beyond PD [34]. Such findings suggested that traditional response criteria may not be able to fully capture the immune-therapy activity. Because of the retrospective nature of this analysis, such results should be interpreted cautiously and need to be confirmed by prospective studies. The immune-related response criteria (ir-RC) have shown to be more appropriate to capture the novel response patterns observed with immunotherapeutic agents and should be included in clinical trials investigating immunotherapies. Indeed according to the ir-RC the appearance of new lesions doesn't necessarily identify a PD, because responses may occur also after a long time and/or a conventional “RECIST” PD [35]. Another limitation of our analysis is represented by the heterogeneity of the selected studies, including four phase I-II single arm studies [20, 21, 26, 30] and three randomized phase II-III clinical trials [23, 24, 27], which investigated different anti-PD1/PD-L1 MoAbs. However, all these MoAbs act on the same immune-modulating pathway, but especially the same study population. Indeed only studies reporting data dedicated to pre-treated NSCLC patients were included, in order to enhance the precision and the accuracy of the results. Finally the high PD-L1 expression heterogeneity in the tumor microenvironment could further reduce the potential role applicability of such predictive biomarker for clinical setting [36]. Indeed PD-L1 expression seems to be a dynamic biomarker, subjected to both space (primary vs metastatic lesions) and time (interval between biopsy and subsequent treatments)-dependent variability [37]. A biopsy sample is just a snapshot of the tumor not reflecting the overall tumor microenvironment. A recent study has shown a low intra-patient heterogeneity and temporal changes in PD-L1 expression using paired synchronous and metachronous tumor specimens of 39 NSCLC patients treated at Memorial Sloan Kettering Cancer Center [38] supporting the reliability of such biomarker for clinical use. In addition to such technical issue, some biological aspects related to the PD-L1 expression need to be also pointed out. Recent evidences suggested two different pathways modulating the PD-L1 expression on the tumor cell surface: the “inflammation-driven”, INF-gamma-mediated, PD-L1 expression, which is localized at sites of inflammation and is usually associated with a baseline tumor T-cell infiltration; and the “oncogene-driven”, PD-L1 expression, which is constitutive, not increased by the inflammation process and associated with neither an immune response nor a T-cell tumor infiltration [39, 40]. Since several studies showed a significant correlation between a baseline T-cell infiltration in the tumor microenvironment and the clinical responses to immune-checkpoint inhibitors [41-43], it may be hypothesized that the first group of tumors, with an inflamed tumor microenvironment, will benefit more from anti-PD-1/PD-L1 directed therapies, compared to those with an oncogene-driven PD-L1 expression status, in absence of T-cell infiltration in the tumor microenvironment. The search for predictive biomarkers needs to be implemented by the identification of other pathological or genomic determinants of response to these therapies. Some studies have recently demonstrated that tumor infiltrating immune cell IHC PD-L1 expression predict responses to Atezolizumab stronger than tumor PD-L1 expression [44], and that combining both tumor-infiltrating lymphocytes and IHC PD-L1 expression status, allow to identify four different subgroup of tumor microenvironment, which could benefit from different treatment strategies, including combination therapies [45]. Another work has shown that an elevated non-synonymous mutation burden, including DNA repair mutations, a molecular smoking signature, and a high neo-antigen load, are strongly associated with clinical activity of Pembrolizumab in NSCLC patients [46]. Some correlations between tumor mutation burden and PD-L1 expression have been also found, even if it needs to be better explored in larger prospective studies.

Despite several limitations our pooled-analysis shows that tumor PD-L1 expression, IHC cut-off >1%, is associated with significantly higher ORRs to anti PD-1/PD-L1 MoAbs, in pre-treated NSCLC patients. These data suggest that PD-L1 at the lowest expression level, though still limited by varied testing approaches and definitions of positivity, has a potential role as predictive biomarker for clinical setting. Probably, as suggested by recent studies, a combination of tumor PD-L1 expression with other “immune-biomarkers” will enhance our ability to identify not only the best candidate to receive immune-therapies, but also the mechanisms of immune-evasion at a single patient level, and ultimately personalize the immune-treatments and combination strategies.

## MATERIALS AND METHODS

### Search for clinical trials

We performed our pooled-analysis according to a predefined written protocol. We searched for all published studies, that report the ORR of patients with NSCLC treated with anti PD1/PD-L1 MoAbs, who received prior chemotherapy regimens, stratified by tumor PD-L1 expression status (IHC cut-off point 1%). Publications were identified by an electronic search in Medline, using PubMed online service, updated in October 2015. The search for publications was made by other databases including the Cochrane Library and EMBASE. However the search on PubMed allowed the widest collection of publications about this topic. The following search terms were used: “PD-1”, “PD-L1”, “lung cancer”, “NSCLC”, “non-small cell lung cancer”, “predictive biomarker”, “anti-PD1”, “anti-PD-L1”, “monoclonal antibodies”, “cancer immunotherapy”. The search was limited to human studies in the English language. The results were supplemented with manual searches of American Society of Clinical Oncology (ASCO), European Society of Medical Oncology (ESMO), and World Conference of Lung Cancer (WCLC), meeting proceedings, references of selected articles and published reviews. A systematic review on this topic in the Cochrane database of systematic reviews was not found.

### Selection criteria

According to this search clinical trials were taken into account if they had to fulfil all the following inclusion criteria: 1) only patients with metastatic NSCLC were included; 2) only patients who received prior therapies were included 3) studies that report the ORR of NSCLC patients treated with an anti-PD1/PD-L1 MoAb as single agent; 4) studies that report the ORR, stratified according to the PD-L1 expression status (IHC cut-off point 1%).

### Data extraction

Two authors independently selected studies according to the aforementioned inclusion criteria. If these two authors could not reach a consensus, another author was consulted and a final decision was made by consensus. Information was carefully extracted by overall selected studies. The following data were collected and organized from eligible studies: first author name, journal and year of publication, study design, study treatment, baseline characteristics of patients (i.e. age, stage, PD-L1 expression status), and ORR stratified according to tumor PD-L1 expression status. The proportion of patients for each outcome was calculated basing on the percentages reported in included trials, when it was not reported as absolute number. Data extraction was conducted according to the Preferred Reporting Items for Systematic Reviews and Meta-Analysis (PRISMA) guidelines [47].

### Statistical analysis

Patients were stratified according to PD-L1 expression status into 2 groups: PD-L1 positive (PD-L1 tumor cell staining ≥1%) and PD-L1 negative. The outcome measure was ORR, defined as the percentage of patients who have a complete or partial tumor response according to World Health Organization (WHO) criteria or Response Evaluation Criteria in Solid Tumors (RECIST), calculated in PD-L1 positive over PD-L1 negative NSCLC patients. The number of events (i.e. objective responses) was extracted from each study or calculated from the percentage provided, and the proportion of patients was calculated for each group. The association between these endpoints and PD-L1 expression status was expressed as an odds ratio (OR) of PD-L1 positive over PD-L1 negative patients. Thus, an OR greater than 1 indicates that PD-L1 overexpression (PD-L1 tumor cell staining ≥1%) is associated with higher ORR in pre-treated NSCLC patients. A pooled-analysis of ORs was performed to calculate a pooled OR for such outcome, using a fixed-effect or random-effect, based on statistical significance of Q-test, according to Mantel-Haenszel method. The heterogeneity between trials was tested using the Cochran Q-test, with a predefined significance threshold of 0.1. Publication bias for ORR analysis were assessed using Begg's funnel plots and Egger's test. The level of significance was set at 5% (P<0.05 suggested a statistical significant publication bias). All statistical analyses were performed with Review Manager 5.3.5 (RevMan; version 5.3.5) and Comprehensive Meta-Analysis software (CMA; version 3.0).
